# Evaluation of Community Resilience in Rural China—Taking Licheng Subdistrict, Guangzhou as an Example

**DOI:** 10.3390/ijerph18115827

**Published:** 2021-05-28

**Authors:** Jianhong Fan, You Mo, Yunnan Cai, Yabo Zhao, Dongchen Su

**Affiliations:** 1 School of Architecture and Urban Planning, Guangdong University of Technology, Guangzhou 510090, China; fanjh7576@gdut.edu.cn (J.F.); 3117008134@mail2.gdut.edu.cn (D.S.); 2 Key Laboratory of Urban Land Resources Monitoring and Simulation, Ministry of Natural Resources, Shenzhen 518034, China; 3 China City Development Academy, Beijing 100009, China; 13794367073@163.com

**Keywords:** resilience, rural community, rural revitalization, social-ecological system, adaptive cycle, sustainability

## Abstract

Resilience of rural communities is becoming increasingly important to contemporary society. In this study we used a quantitative method to measure the resilience regulating ability of rural communities close to urban areas—in Licheng Subdistrict, Guangzhou City, China. The main results are as follows: (1) Rural systems close to urban areas display superior adapting and learning abilities and have a stronger overall resilience strength, the spatial distribution of which is characterized by dispersion in whole and aggregation in part; (2) the resilience of most rural economic subsystems can reach moderate or higher levels with apparent spatial agglomeration, whilst the ecological subsystem resilience and social resilience are generally weaker; the spatial distribution of the former shows a greater regional difference while the latter is in a layered layout; (3) some strategies such as rebuilding a stable ecological pattern, making use of urban resources and cultivating rural subjectivity are proposed on this basis, in order to promote the sustainable development of rural areas and realize rural revitalization. This work also gives suggestion for the creation of appropriate and effective resilience standards specifically targeted for rural community-aiming to achieve the delivery of local sustainability goals.

## 1. Introduction

Resilience, especially community resilience, is a function of an ecosystem’s absolute ability to recover from various stresses and disturbances [1], particularly in relation to resisting shocks, economic downturns, climate change, globalization and environmental disasters [2,3]. The resilience of the community system; therefore, is not only a reflection of the pressure resistance, adaptability and innovation of the rural system but also is one of the breakthrough points, or at least a major turning point for current research into urban and rural development in China [4,5]. The rural community results from the coupling between people and the natural environment [6]. The internal structure and function of the rural community maintains a relatively balanced state depending on the resilience of the system and is reflected in economic, ecological and social subsystems [7]. The rural system is mainly composed of three subsystems: ecological, economic, and social [8]. Among them, the ecological subsystem resilience is mainly reflected in the stock of cultivated land, the coverage of forest and the storage capabilities of hilly ponds (fish ponds formed under natural low hill terrain) [9]. Furthermore, the economic subsystem resilience is generally measured using the per capita income of farmers and the yield of economic crops [10]; whilst that of the social subsystem is based on the rural social network and is a reflection of the rural people-land relationship [11]; thus, the social subsystem is significantly affected by the mobility of people, especially from rural villages.

Resilience theory, which is at the core of the research reported in this paper, was originally employed to study the anti-interference ability of ecosystems [12,13,14,15], in other words, the maximum tolerance within which an ecosystem can maintain its stable state when facing change. In the 1970s, Gunderson and Holling introduced the resilience concept into social ecological systems. Holling stated that, resilience is the ability of a system to resist interference and maintain its function and control (i.e., the interference level that the system can resist while maintaining its function or the measure of capacity that the ecosystem absorbs the change and maintains its state), and further stated that, such a resilient system can effectively deal with the uncertainty that it could face in the future through the resilience of its management system [16,17,18]. As a comprehensive reflection of social, economic and ecological subsystems etc. in time and space, the rural community system has specific characteristics such as complexity, ability to self-organize, and diversity. With an increasingly close relationship and increasingly apparent mutual influence between people and nature, studying the response and adaptability of the system (with the social ecological system as the research object) to external interference from the perspective of resilience has recently attracted the attention of sustainable development research [19,20,21,22]. The prominent international academic organization “Resilience Alliance”, led by Holling, used the theory of adaptive cycle to describe and analyze the dynamic mechanism of the social ecological system and put forward that such a system will successively go through four stages, namely development, protection, release, and update. These development stages constitute an adaptive cycle based on certain rules [23,24,25,26,27]. In the development phase, the system has just formed, and its resilience is gradually improved by continuously absorbing elements and establishing the connection between elements. In the protection phase, the system gradually developed, with the potential for growth decreasing and resilience weakening. In the release phase, the system is affected by a large number of interference factors and becomes disordered. At this time, system’s resilience is low. Interference factors have some ruinous capacity to destroy the system and are the sources of the update phase. The system makes use of its resilience to obtain an opportunity of reorganization by learning and adapting to changes, and then realizes reorganization. Then, the system goes into the development stage again and goes back and forth, or the system collapses due to the lack of enough resilience, so as to exit the cycle [24,25,27].

In related research, the large-scale rapid outbreak of interference factors, such as the Sichuan earthquake in 2008, can serve to arouse the research interest of researchers and, consequently, the associated literature also appears in this stage [28,29,30]. However, in recent years, for the rapid and accidental external shock events, there has emerged an active interest in the adaptation and cultivation of the concept of resilience. Resilience research has shifted to the study of chronic and complex factors, with more emphasis on active human agency, using the “bottom-up” community-led development model [14,31,32]. In terms of research content, there are an increasing number of studies on the resilience of rural communities. Some scholars investigated the resilience of rural agriculture in the “agricultural” and “non-agricultural” processes, and concluded that resilience is driven by internal and external agricultural processes [33]. Furthermore, some scholars have formulated normative requirements and practical solutions of rural resilience development on the basis of known influencing factors of resilience, and connected the theory to practice using the community resilience framework [34,35]. The rural policy conducts critical research and proposes normative requirements regarding the ability of digital technology to help rural development, the provision of rural service solutions and the challenges of implementing localism [36]. In terms of research methods, scholars have used frameworks to assess the resilience of rural communities. For example, Jurjonas and Seekamp [37] proposed the rural coastal community resilience (RCCR) framework which include maintaining rural livelihoods, creating job opportunities, and addressing highly vulnerable populations in eastern North Carolina; Wilson [18] built on a conceptual framework to assess community resilience in rural China, and argued that Chinese government policies need to be substantially realigned if the resilience of rural communities is to be improved.

Research on resilience theory has moved on from discussions of concept connotation and construction of a theoretical framework to the establishment of a resilience evaluation system [38]. However, despite there being many frameworks for evaluating community resilience using qualitative analysis, currently, only a few frameworks use a quantitative approach in their research. In addition, in the context of urban-rural integration and rural revitalization proposed by China, the existing community resilience evaluation framework has a relatively small scope of application, and there is no research on the evaluation of the resilience of urban fringe villages; furthermore, the research on system resilience function evaluation is relatively deficient due to the complexity and diversity of social ecological systems [39]. The aim of the research in this paper; therefore, is the measurement of the resilience regulating ability of rural communities close to the urban areas by constructing a new index system for the evaluation of social-ecological system resilience, thus developing a quantitative research approach, which combines the visualization function of the Geographical Information System (GIS) to reveal the resilience distribution characteristics of various subsystems on a village scale in order to provide a new research perspective for the development and revitalization of rural communities.

## 2. Materials and Methods

### 2.1. Study Area

**Figure 1:**
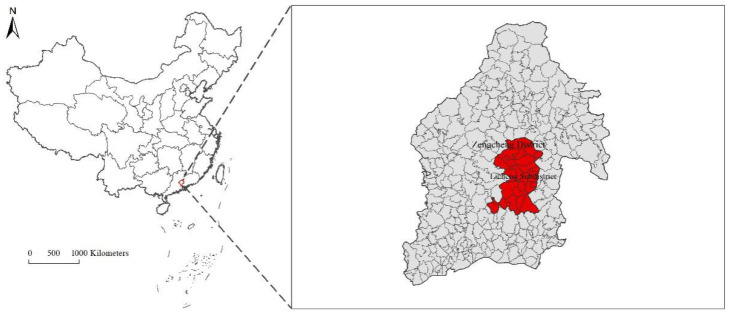
Location of the research area.

**Figure 2:**
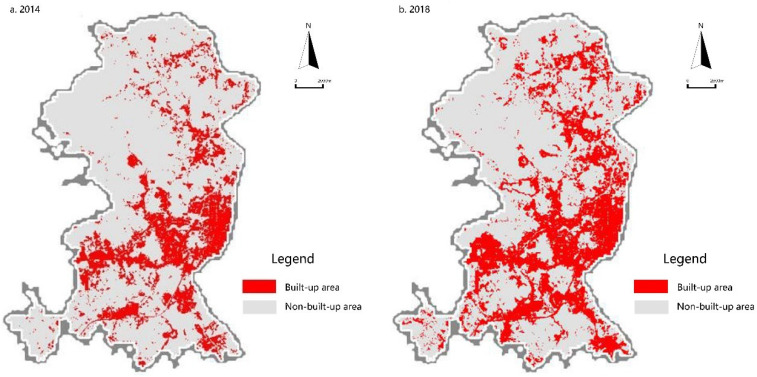
Growth of the built-up areas in Licheng Subdistrict from 2014 to 2018.

Zengcheng District is situated in the eastern part of Guangzhou City, which is located in central Guangdong Province to the south of China (Figure 1). After Zengcheng was annexed to Guangzhou as a district in 2014, in order to comply with the urban division adjustment of Guangzhou, Guangzhou Municipal Government produced the eastward development planning strategy and gradually transferred superior urbanization resources (such as the input of pillar industrial resources and structures) that accumulated over the years in Zengcheng District. As a core economic development area, Licheng Subdistrict became a district with prominent ecological, economic and social contradiction problems. In this paper, the remote sensing images of Licheng Subdistrict, taken in 2014 and 2018 were processed and an analysis of the characteristics of changes in the size of the built-up area was carried out. A comparison of the built-up area (Figure 2) in 2014 and 2018 shows an obvious trend of expansion that is both typical and representative. For these main reasons, the 24 administrative villages in the Licheng Subdistrict of Zengcheng District were chosen as the research object for this paper.

The rural community system, which is located in the core urban development zone, is an area of centralized exchange and interchange of urban-rural elements and presents a distinct interference landscape variation phenomenon on economic, ecological and social subsystems. The massive influx of new urban resources effectively broke through the interface of traditional rural communities and forced rural grassroot communities to turn from closure to opening-up. The resilience of rural communities; therefore, is faced with unprecedented challenges.

### 2.2. Data Source

The research underpinning this paper takes all of the administrative villages (24) in the Licheng Subdistrict as evaluation units; uses basic village data from the latest statistics sourced from the town government of Licheng Subdistrict together with the 2016 village data provided by the Zengcheng Urban and Rural Planning Design and Research Institute (Table 1). However, due to the Zengcheng Gualv Lake water conservation project, the residents of the villages of Luogang, Mingxing, Taiping, Guangming and Xigualing were relocated which meant that these villages no longer had the attribute of a traditional rural area. Therefore, the basic data pertaining to these five villages were ignored, leaving the remaining 19 administrative villages as the research object.

### 2.3. Research Methods

#### 2.3.1. Construction of the Index System

This research takes the economic subsystem resilience, ecological subsystem resilience and social subsystem resilience as primary indexes and designates secondary indexes according to the attribute features of the subsystems to construct the rural community resilience function evaluation index system. The characteristics of changes in geographical and human landscapes that the ecological, economic, and social subsystems show under the interference from urbanization are considered comprehensively for index selection and are quantified for the designation of secondary indexes (Table 2). Through the three primary indexes and eight secondary indexes, the resilience index system is constructed to analyze the resilience function of the rural community system for Licheng Subdistrict. Indexes that help strengthen the ecological environment, economic income and social stability are positive indexes. The higher in magnitude of a positive index value implies that the resilience is stronger. On the contrary, indexes that are bad for the development of the rural ecological social system are negative indexes. The higher in magnitude negative index value implies that the resilience is weaker.

#### 2.3.2. Data Standardization and the Determination of Index Weight

For the standardized processing of the original data, this paper adopts the range method as expressed in Equation (1):

(1) $$Y_{ij}=\{\begin{array}{c} \left(X_{ij}-X_{min}\right)/\left(X_{max}-X_{min}\right)\\ \left(X_{max}-X_{xj}\right)/\left(X_{max}-X_{min}\right) \end{array}$$

In Equation (1), *Y_ij_* is the standardized value of the index in the year; *X_xj_* is the original value of the index, *X_max_* and *X_min_* are the maximum and minimum values of the index, respectively.

The entropy weight method is used to determine the weight [40,41], so as to effectively avoid any influence of the expert’s subjective judgment errors on the weight analysis and, thus, to make the evaluation result more objective. The various calculations are as follows:

Equation (2) is used to calculate the proportion of Index *j* in Year *i*

(2) $$P_{ij}=\frac{Y_{ij}}{\sum_{i=1}^{m}Y_{ij}}$$

Equation (3) is used to calculate the entropy of Index *i*:

(3) $$e_{j}=-k\sum_{i=1}^{m}\left(P_{ij}\times \ln P_{ij}\right)$$

Equations (4) and (5) are used to calculate the weight of each index based on the result from Equation (3):

(4) $$g_{j}=1-e_{j}$$

(5) $$w_{j}=g_{j}/\sum_{i=1}^{m}e_{j}$$

Equation (6) is used to calculate the resilience function value for the subsystems of each administrative village:

(6) $$Z_{ij}=w_{j}\times Y_{ij}$$

Equation (7) is used to calculate the total system resilience value for each administrative village:

(7) $$S_{ij}=Z_{ij}+Z\prime_{ij}+Z{\prime \prime }_{ij}$$

**Figure 3:**
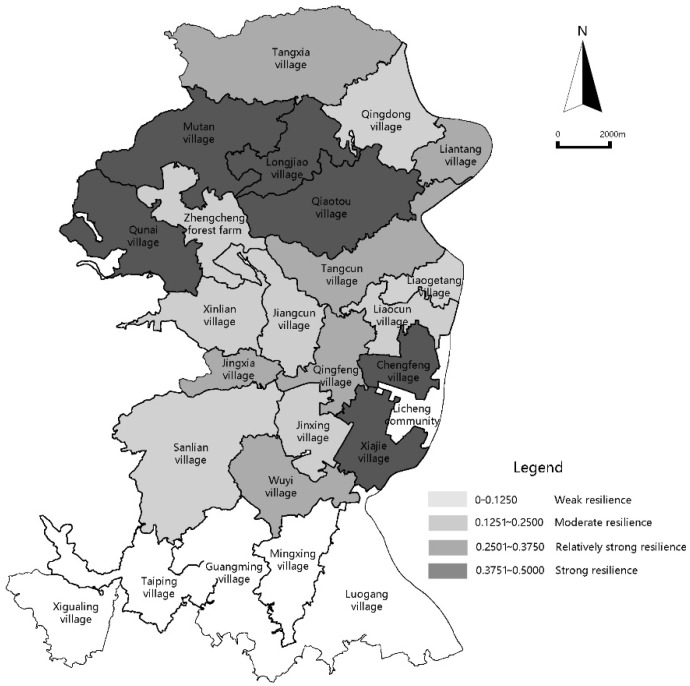
Diagram of rural system overall resilience function values.

The data for the administrative villages of Licheng Subdistrict after standardized processing are listed in Table 3. Using the entropy method as detailed in Section 2.3.2, the weights for the various indexes were determined and used in the calculation of the resilience function measurement results, which are listed in Table 4. The visual resilience function values determined using the GIS Nature Breaks method are shown in Table 4 and Figure 3.

## 3. Results

### 3.1. Rural System Resilience Strength and Spatial Distribution Characteristics

#### 3.1.1. Rural System Resilience Strength

Figure 3 is the rural community system overall resilience function values, from which it can be seen that Licheng Subdistrict is in the value range 0.1462–0.4647, a moderate resilience state. According to the classification standard from relevant research [42,43], resilience strength can be divided into four grades: The strong value range is 0.3751–0.5000; the relatively strong value range is 0.2501–0.3750; the moderate value range is 0.1251–0.2500; the weak value range is 0.0000–0.1250. No village in Licheng Subdistrict has yet fallen into the weak value range. However, five villages fall into the strong range and five villages fall into the moderate range, accounting for 31.58% of the total. Ten villages fall into the relatively strong range, accounting for 47.37% of the total, the highest proportion. It can be found that most villages in Licheng Subdistrict have good adapting and learning abilities in addition to a good regulation ability.

#### 3.1.2. Rural System Resilience Spatial Distribution Characteristics

The spatial distribution of the resilience strength of administrative villages in Licheng Subdistrict is characterized by dispersion in whole and aggregation in part. This means that villages with different resilience levels are distributed dispersedly, but villages that fall into the strong value range are aggregated in northern central areas (such as Mutan village, Longjiao village, Qiaotou village and Qunai village) and some eastern areas (such as Chengfeng village, Xiajie village). It can be seen that villages with strong resilience are characterized by linkage and sprawl, which helps northern and central regions of Licheng Subdistrict absorb urban resources and promote rural revitalization.

### 3.2. Strength and Spatial Distribution Characteristics of Resilience of Rural Subsystems

#### 3.2.1. Ecological Subsystems

**Figure 4:**
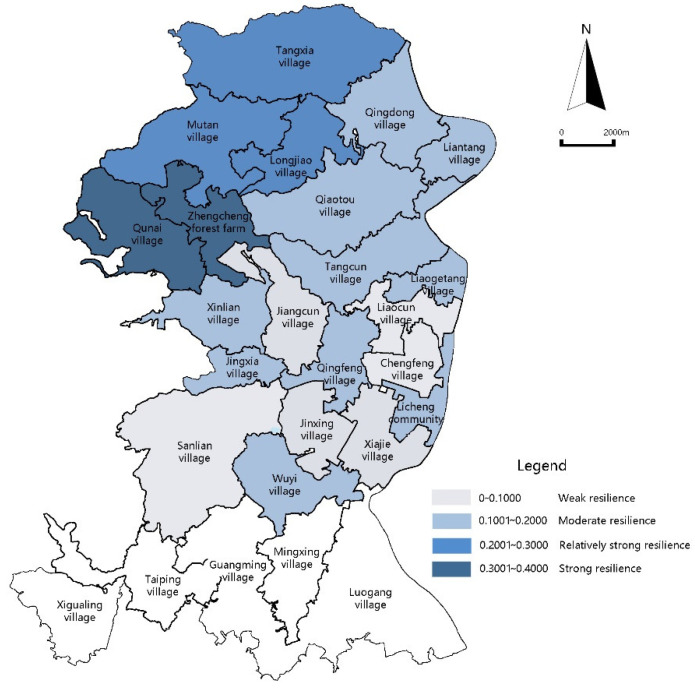
Diagram of ecological subsystem resilience function values.

For the resilience function values of the ecological subsystems for Licheng Subdistrict, the strong value range is 0.3001–0.4000; the relatively strong value range is 0.2001–0.3000; the moderate value range is 0.1001–0.2000; and the weak value range is 0–0.1000. The resilience function values of the ecological subsystems for most villages in Licheng Subdistrict fall into the moderate and weak value ranges. There are 15 administrative village units in this range, accounting for 78.9% of the total. It can be seen in Figure 4 that the ecological subsystems for Licheng Subdistrict lack resilience and the ecological environment is generally highly fragile.

The ecological environment is closely related to a good agricultural foundation. The strong resilience value range of the ecological subsystems is centralized in the villages of Qun’ai, Tangxia, Mutan, and Longjiao in the northern part of Licheng Subdistrict, which is the main production area for large scale agricultural units, enterprises, farming cooperatives and production bases of the wider Zengcheng District. Adopting completed ecological environment protection measures, Qun’ai Village is the main cultivation base of mesona chinensis benth (one of the “Ten Treasures in Zengcheng”). The central area basically falls into the low resilience function value range and an aggregation block with Xiajie Village in the center is formed.

#### 3.2.2. Economic Subsystems

**Figure 5:**
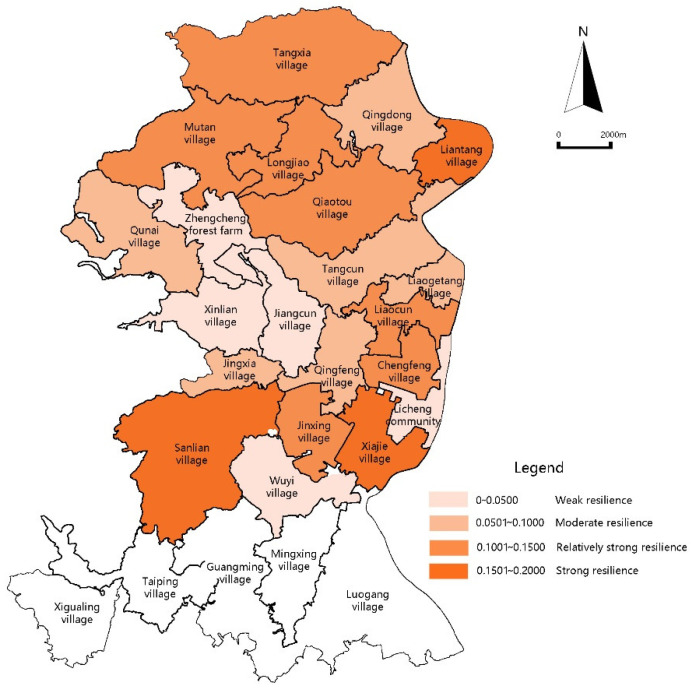
Diagram of economic subsystem resilience function values.

The function values of the economic subsystems for Licheng Subdistrict are in the range 0.010–0.200, implying a relatively strong overall resilience function with 84.21% of the administrative villages reaching moderate or higher levels. However, the function values show a large span, and the resilience function ranges of the economic subsystems differ significantly, as can be seen in Figure 5. For instance, the strong economic resilience function value range is located in the central part of the subdistrict, mainly including Xiajie Village and Sanlian Village. Whereas the relatively strong function value range is located in the northernmost part of the subdistrict (i.e., the Tangsha Village-centered continuous rural community); the centrally distributed moderate function value range is located between the strong value range and relatively strong value range.

Comparing the villages of Xiajie, Sanlian and Liantang, which are all in the strong economic resilience function value range, it is found that the annual agricultural income of Sanlian is more than RMB 20 million, whilst Xiajie and Liantang have almost no agricultural income but the per capita income of farmers in these villages ranks top as most of their incomes come from rent and the ecological leisure tourism industry. Tourism matching industries, such as farm tourism and native products, are developed by relying on the unique ecological resources. Rural economic development no longer is based on a single agricultural production methodology but has evolved towards diversified production modes.

#### 3.2.3. Social Subsystems

**Figure 6:**
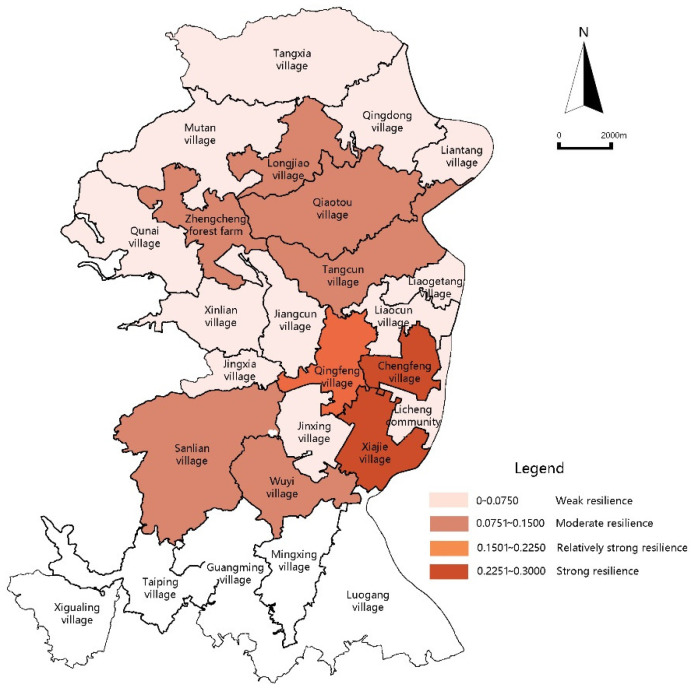
Diagram of social subsystem resilience function values.

The resilience values of the social subsystems for Licheng Subdistrict are obviously weak in the whole and are mostly centralized in the low value range of 0.0000–0.0750. Villages with the weakest social subsystem resilience values are in a zonal distribution and in a layered layout around villages with higher economic levels (Figure 6). Secondary indexes of social subsystems are obtained through quantitative processing and analysis of the situation of rural population loss, talent introduction efforts and cultural heritage protection.

Rural social resilience is based on the connection degree and social familiarity of population settlements (i.e., the acquaintance society network) [44,45]. Since the 1990s, after Guangzhou entered into the stage of high-speed urbanization, villages close to cities were brought to the urbanization system resulting in a strong economic pull factor that further attracted the labor force from less developed surrounding villages. The frequent population flows in rural communities; therefore, increased the heterogeneity of these communities [46,47]. Under such unstable conditions, the stability of existing social relations in rural communities and the knowledge of rural subjectivity are especially critical for the promotion of system resilience. For example, in Xiajie Village, which is in the strong social resilience value range, there are many cultural heritage assets such as the Xiajie Ancient Post Road and Huaiyin Hall. Strengthening cultural awareness can contribute to the cohesion of communities. In addition, Xiajie Village attaches great importance to talent introduction. Aiming to protect traditional ancient villages and restore rural vitality, the Ancient Village Friendship Association of Xiajie Village has assembled a team of professional planners who are proud of their native land and are actively participating in events to promote rural revitalization. Besides, activities periodically held in Xiajie Village provide consultation services for migrant workers including health examinations and parent-child communication activities to enable migrants who live there to develop a sense of belonging to Xiajie Village and consciously adhere to its rules and regulations. This continuously helps to promote rural social resilience.

## 4. Discussion

**Figure 7:**
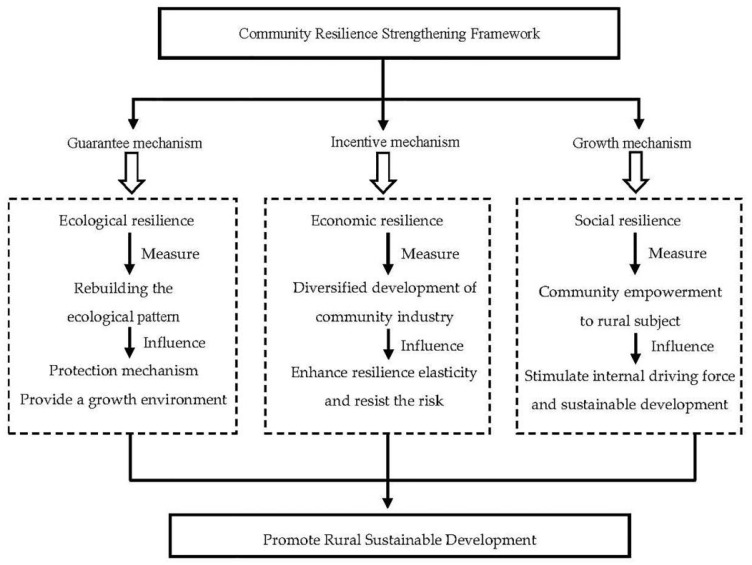
Diagram of the rural community system resilience improvement framework.

Based on the calculation results above, it can be concluded that rural communities in Licheng Subdistrict have a good overall resilience, but the development of subsystems is extremely unbalanced and there are many problems that need to be solved. Villages close to the core urban development area have a relatively strong overall resilience, good adapting and learning abilities that can generally meet the basic living demands of villagers. The resilience of subsystems, however, is unbalanced. Although the resilience of economic subsystems is relatively strong, the resilience of social and ecological subsystems differ significantly and the overall situation; therefore, is not optimistic. This is especially the case concerning the resilience of the ecological subsystem, which is apparently weakened against the long-term development of rural communities. The diversified economic development modes provide an appreciation space for villages to attract the inflow of capital. However, a destructive impact has been made on the ecological environment due to the imperfect rural market mechanism, which is not conducive to the stable development of the rural ecological social system. Currently, rural communities in the subdistrict are in the phase of high-speed transformation of urban-rural elements. In the process of urban sprawl, traditional rural life, dominated by farming culture with blood-tied relationships, has been eroded by modern culture. New planning, design, and construction achievements are separated from the original cultural landscape planning of traditional villages. The conflict between traditional civilization and modern civilization is exactly a reflection of the rural community system resilience being subjected to “Strong Interference”. The research carries out index quantification for such an “Interference Degree”, measures it through the threshold range, explores the strength of the overall resilience function of rural communities under the interference of urbanization through the combination of both qualitative and quantitative methods, and provides strategies for strengthening community resilience according to the analysis results (Figure 7).

The strong value range of rural system resilience is mainly distributed in the northern region and partially centralized in the central region. Due to the differences in the abilities of the economic, social, and ecological systems to cope with interference, the resilience of each subsystem has different spatial distribution characteristics. The ecological subsystem has an obvious polarization in resilience. The northern region comprises many modern agricultural bases which have better soil and water conservation capacities. Therefore, the ecological system in the northern region is less damaged. Through spot investigation, it is found that Chengfeng, Jinxing, Qingfeng, and Wuyi around Xiajie are all urban villages. Agricultural production has seceded from the main local economic structure. Working outside and leasing houses for rent are the main means of livelihood of the villagers. As the earliest core area that takes over the outflows of urban resources, the central area of Licheng Subdistrict is undergoing increasingly severe urban sprawl that is causing a significant decrease in cultivated land which further causes a reduction of the eco-environmental quality in rural areas and directly affects the resilience of the local rural ecological system. Rebuilding a stable ecological pattern is critical for the current weak rural ecological subsystem resilience function. The original ecological bases of most villages were gradually changed and original farming landscapes were devoured by industrial landscapes under the trend of urban sprawl. The blurring of urban and rural landscape boundaries and the integration of rural spatial features are serious problems in the current rural development process. Restoring the rural landscape is the basis for a good system cycle. To reshape the ecological pattern, the coordination mechanism between urbanization and ecological environment should be established. External tension focuses on strengthening population quality and legal norms, technological innovation, and industrial upgrading, while internal tension depends on residents’ awareness of environmental protection.

The enhancement in the resilience function values of the rural economic subsystems for Licheng Subdistrict can be attributed to an influx of new activities during the process of urbanization. These activities, such as the rental of houses, ecological leisure tourism, and industrial development, have created a favorable environment for structural and functional reorganization of the system, so as to adapt the systems to the urbanization development mode. Loreau et al. stated the importance of species richness of the system [48]. The immigration of alien species can improve the adaptability of the ecological system to changes, causing a fundamental reaction of the ecological system and in so doing, changing its performance characteristics. The accumulation of adaptability allows for improvements in system resilience [49,50]. Rural economic development no longer is based on a single agricultural production methodology but has evolved towards diversified production modes. This is consistent with the theoretical analysis on the mutual influence between biodiversity and resilience [51]. In view of the high average economic system resilience of rural communities in Licheng Subdistrict, villages need to use their own resources to find a breakthrough in the market, change the role of passive recipients, and take the initiative to use diversified urban resources to improve the creativity of the system. The capital investment frenzy caused by urbanization has become an irresistible trend. If the resilience of rural economic systems cannot actively filter and absorb the outflow of resources, it will inevitably be swallowed up by market competition and fall victim to the capital cycle. Therefore, when facing capital invasion, the capital opportunity of the market can be utilized to enhance the resistance ability of the economic system and adapt to its competitive and changeable interference. The sustainable development of rural areas cannot rely on a single industry. The current technological developments have greatly reduced the manpower requirements for agricultural production, and the redundant human resources need more employment opportunities to make a living. The diversified economic development mode can not only solve the problem of a surplus of farmers, but also reduce the centralized destruction of interference sources.

It is through the change of economy and ecology that has changed the original rural social relations. In order to pursue employment opportunities and a good living environment, most villagers flow to areas with higher economic levels and more comfortable ecological environments. The rural population flow has the characteristic of selecting the nearest optimal location, which is reflected in the layered decline of the resilience of the social subsystem, forming a typical core-edge structure. The optimization strategy for the resilience function of the social subsystem of rural areas can start from the cultivation of rural subjectivity [26]. The arrival of the so-called global competition age, served to interfere with the endogenous development of rural communities which were essentially squeezed by a more powerful foreign culture, resulting in those rural communities losing the internal driving force of development and autonomy. A prominent manifestation of this is the massive loss of villagers from several villages in Licheng Subdistrict and the inflow of a foreign population. Therefore, it follows that strengthening the subjectivity of rural communities is especially important for managing “non-acquaintance society” relations. With a long history, the villages in Licheng Subdistrict were mostly built in the Qing Dynasty. Many ancestral halls are set up and the profound ancestral idea foundation is kept in these villages. At the same time, showing significant cultural potential for the whole subdistrict, there is one historical and cultural site protected at the provincial level, two sites are protected at the municipal level, and 33 sites protected at the district level (36 in total). Therefore, in respect of the strategy of adjusting the resilience of the rural social subsystem, the rich cultural resources can be used to cultivate the cultural identity of villagers, reshape the human network of the social system, and strengthen the regulating ability of social resilience. For example, Licheng Subdistrict has responded positively to the construction of the cultural line of the ancient post road carried out in the last two years. Professionals in planning, local elites and the government have cooperated actively to carry out investigations and obtain on the spot evidence so as to arouse the villagers’ recognition of their own culture imperceptibly. In addition, social activities such as the post road activity also promote the regeneration of the rural social system.

## 5. Conclusions

The aim of the study was to assess the resilience regulating ability of rural communities by constructing a new index system for the evaluation of social-ecological system resilience. This approach contrasts with most other resilience evaluation frameworks that are based on qualitative analysis, for example, using NVivo (QSR International, Melbourne, Australia) to analyze data obtained through semi-structured interviews [52]. The quantitative approach using the new index essentially contributes to broadening the scope of resilience measurement research and appears to be especially beneficial in the analysis of rural areas that are close to urban areas. We also analyzed the evolution mechanism and driving mechanism of rural communities from the perspective of protection and development strategy in the new dimension of resilience, and explored a new perspective for rural rejuvenation in practice. The main contributions of this paper are as follows: (1) Rural systems close to urban areas have a stronger resilience strength, its spatial distribution shows dispersion in whole and aggregation in part. (2) The resilience of most rural economic subsystems are higher, while the ecological and social subsystems are generally weaker. (3) In addition, we proposed some policy suggestions to promote the sustainable development of rural areas and realize rural revitalization.

The influences of multiple factors need to be considered for the establishment of the resilience index system, which for the research area selected in this paper may have some limitations in the determination of the rural community resilience function values due to limited data. More comprehensive data indexes, such as the ratio of agricultural income and the ratio of secondary and tertiary industries, can be considered in subsequent research. In addition to the expansion of objective data, on-the-spot tracking investigations can be enhanced to verify the accuracy of the results in practice. The future planning should not only optimize the material space, but also use a comprehensive perspective to analyze the rules of the rural social ecological system, stimulate the driving mechanism of rural development, and encourage more villages to move towards a new phase of growth and protection.

## Figures and Tables

**Table 1:** Data source.

Dataset	Data Description	Data Source
Administrative division data	Shapefile, in 2018	Guangzhou Municipal Planning and Natural Resources Bureau (Zengcheng Branch)
Socio-economic statistical data	Excel, in 2018, village as the basic unit	Licheng Subdistrict Office
Census data	Excel, in 2018, household registered population, migrant workers, party members	Licheng Subdistrict Office
Cultural relics volume	Excel, in 2018, village as the basic unit	Licheng Subdistrict Office
Cultivated land, forest	Excel, in 2018, village as the basic unit	Licheng Subdistrict Office
land, ponds, yield of economic crops	Excel, in 2018, village as the basic unit	Village Committees in Licheng Subdistrict

**Table 2:** Rural social-ecological system resilience function evaluation index system.

Primary Indexes	Secondary Indexes	Index Description	Attribute
Ecological subsystem resilience	Area proportion of cultivated land	Area of cultivated land/area of village	*x*_1_ positive index
	Area proportion of forest land	Area of forest land/area of village	*x*_2_ positive index
	Area proportion of ponds	Area of ponds/area of village	*x*_3_ positive index
Economic subsystem resilience	Proportion of per capita income of farmers	Per capita income of farmers in the villages/Per capita income of farmers in the whole subdistrict	*x*_4_ positive index
	Yield of economic crops	Yield of economic crops in the villages/total yield of economic crops	*x*_5_ positive index
	Proportion of Party members	Number of Party members in the villages/total number of people of the villages	*x*_6_ positive index
Social subsystem resilience	Proportion of migrant workers	Number of migrant workers/total number of people of the villages	*x*_7_ negative index
	Proportion of cultural relics	Number of cultural relics in the villages/number of cultural relics in the whole subdistrict	*x*_8_ positive index

**Table 3:** Standardization results of indexes of resilience function of Licheng Subdistrict administrative village system.

Name	*x*_1_	*x*_2_	*x*_3_	*x*_4_	*x*_5_	*x*_6_	*x*_7_	*x*_8_
Sanlian Village	0.1781	0.1429	0.2000	0.1612	0.1875	0.4000	0.6786	0.3000
Wuyi Village	0.0000	0.7143	0.2000	0.1208	0.0000	0.4000	1.0000	0.1500
Xiajie Village	0.0000	0.0000	0.2000	0.6309	0.0625	0.8000	0.1429	1.0000
Chengfeng Village	0.0959	0.0000	0.6000	0.3490	0.1250	0.4000	0.0000	1.0000
Liaocun Village	0.7535	0.0000	0.2000	0.2416	0.3125	0.6000	0.6423	0.0000
Getang Village	1.000	0.0000	0.4000	0.1544	0.2500	0.6000	0.2143	0.0000
Tangcun Village	0.4795	0.0000	0.8000	0.0537	0.3125	0.2000	0.9643	0.3000
Qiaotou Village	0.3699	0.2857	0.6000	0.1074	1.0000	0.2000	0.9642	0.1500
Longjiao Village	0.3288	0.4285	1.0000	0.0805	0.8750	0.4000	0.7143	0.1500
Mutan Village	0.5068	1.0000	0.6000	0.0671	1.0000	0.2000	0.5357	0.0000
Qingdong Village	0.5890	0.1429	0.6000	0.0738	0.3125	0.2000	0.8571	0.0000
Tangsha Village	0.2877	0.5714	0.4000	0.1006	0.2500	0.0000	0.1786	0.1500
Liantang Village	0.3425	0.1429	0.6000	1.0000	0.1250	0.4000	0.3929	0.0000
Jinxing Village	0.0000	0.0000	0.0800	0.6578	0.0188	0.4000	0.5357	0.0000
Xinlian Village	0.4247	0.2857	0.0800	0.0872	0.1250	0.4000	0.9642	0.0000
Qingfeng Village	0.1233	0.1429	0.8000	0.0671	0.2500	0.6000	0.8928	0.4000
Jiangcun Village	0.1370	0.0000	0.0800	0.1208	0.1875	0.6000	0.4286	0.1500
Jingxia Village	0.1644	0.4286	0.4000	0.1477	0.2500	1.0000	0.6071	0.0000
Qun’ai Village	0.3972	1.0000	0.1000	0.0000	0.6875	0.6000	0.4643	0.1500

**Table 4:** Resilience function of Licheng Subdistrict administrative village system.

Name	Total Resilience	Ecological Resilience	Economic Resilience	Social Resilience
Sanlian Village	0.2367	0.0640	0.0451	0.1276
Wuyi Village	0.2845	0.1620	0.0177	0.1048
Xiajie Village	0.3986	0.0100	0.1003	0.2989
Chengfeng Village	0.3987	0.0593	0.0655	0.2738
Liaocun Village	0.2240	0.0946	0.0712	0.0583
Liaogetang Village	0.2254	0.1366	0.0512	.0376
Tangcun Village	0.2915	0.1156	0.0436	0.1324
Qiaotou Village	0.3699	0.1459	0.1300	0.0940
Longjiao Village	0.4065	0.2037	0.1118	0.0910
Mutan Village	0.4647	0.3056	0.1241	0.0350
Qingdong Village	0.2366	0.1395	0.0465	0.0505
Tangsha Village	0.2694	0.1791	0.0433	0.0470
Liantang Village	0.3121	0.1140	0.1610	0.0371
Jinxing Village	0.1493	0.0066	0.0987	0.0440
Xinlian Village	0.2006	0.1088	0.0271	0.0647
Qingfeng Village	0.3188	0.1077	0.0384	0.1726
Jiangcun Village	0.1462	0.0208	0.0391	0.0863
Jingxia Village	0.2622	0.1373	0.0502	0.0747
Qun’ai Village	0.4196	0.2531	0.0785	0.0880

## Data Availability

Not applicable.
